# Supplementary material for: Curved adhesions mediate cell attachment to soft matrix fibres in three dimensions
Source: Nat Cell Biol. 2023 Sep 28;25(10):1453–64. doi: 10.1038/s41556-023-01238-1 (PMC10567576; doi:10.1038/s41556-023-01238-1)
Supplement: Supplementary file 1 — Reporting Summary [file 41556_2023_1238_MOESM1_ESM.pdf]

nature portfolio

Corresponding author(s): Bianxiao Cui

Last updated by author(s): Aug 10, 2023

Reporting Summary

Nature Portfolio wishes to improve the reproducibility of the work that we publish. This form provides structure for consistency and transparency in reporting. For further information on Nature Portfolio policies, see our Editorial Policies and the Editorial Policy Checklist.

Statistics

For all statistical analyses, confirm that the following items are present in the figure legend, table legend, main text, or Methods section.

n/a	Confirmed	
☐	☒	The exact sample size (n) for each experimental group/condition, given as a discrete number and unit of measurement
☐	☒	A statement on whether measurements were taken from distinct samples or whether the same sample was measured repeatedly
☐	☒	The statistical test(s) used AND whether they are one- or two-sided<br>*Only common tests should be described solely by name; describe more complex techniques in the Methods section.*
☐	☒	A description of all covariates tested
☐	☒	A description of any assumptions or corrections, such as tests of normality and adjustment for multiple comparisons
☐	☒	A full description of the statistical parameters including central tendency (e.g. means) or other basic estimates (e.g. regression coefficient) AND variation (e.g. standard deviation) or associated estimates of uncertainty (e.g. confidence intervals)
☐	☒	For null hypothesis testing, the test statistic (e.g. F, t, r) with confidence intervals, effect sizes, degrees of freedom and P value noted<br>*Give P values as exact values whenever suitable.*
☒	☐	For Bayesian analysis, information on the choice of priors and Markov chain Monte Carlo settings
☒	☐	For hierarchical and complex designs, identification of the appropriate level for tests and full reporting of outcomes
☐	☒	Estimates of effect sizes (e.g. Cohen's d, Pearson's r), indicating how they were calculated

Our web collection on statistics for biologists contains articles on many of the points above.

Software and code

Policy information about availability of computer code

Data collection	Nikon NIS-Elements AR (updated routinely by the manufacturer), MetaMorph 4.8.3, Leica LAS X (updated routinely by the manufacturer)
Data analysis	MATLAB 2023a and 2017a (MathWorks), ImageJ (Fiji 2.13), Prism 9.5.1 (GraphPad software), and custom MATLAB codes which have been deposited to github (Link: github.com/wzhang5publication/Data-analysis).

For manuscripts utilizing custom algorithms or software that are central to the research but not yet described in published literature, software must be made available to editors and reviewers. We strongly encourage code deposition in a community repository (e.g. GitHub). See the Nature Portfolio guidelines for submitting code & software for further information.

Data

Policy information about availability of data

All manuscripts must include a data availability statement. This statement should provide the following information, where applicable:

- Accession codes, unique identifiers, or web links for publicly available datasets
- A description of any restrictions on data availability
- For clinical datasets or third party data, please ensure that the statement adheres to our policy

Source data are provided with this paper. All other data supporting the findings of this study are available from the corresponding author upon reasonable request. Plasmids originally generated in this study and their sequence information are available at Addgene (IDs: 205090, 205091, 205092, 205093, 205094 and 205095). The expression level of integrins in U2OS cells was obtained from the Human Protein Atlas project's RNA HPA cell line gene data available from v23.0.proteinatlas.org.

March 2021

Field-specific reporting

Please select the one below that is the best fit for your research. If you are not sure, read the appropriate sections before making your selection.

☒ Life sciences ☐ Behavioural & social sciences ☐ Ecological, evolutionary & environmental sciences

For a reference copy of the document with all sections, see nature.com/documents/nr-reporting-summary-flat.pdf

Life sciences study design

All studies must disclose on these points even when the disclosure is negative.

Sample size	No sample size calculation was performed. Sample size was determined based on our previous experimental experience (e.g., Zhao, W. et al. Nat. Nanotechnol. 12, 750–756 (2017); Lou, H.-Y. et al. PNAS 116, 23143–23151 (2019)). Similar sample sizes have been used in many recent papers from other labs, such as Shiu, J.-Y. et al. Nat. Cell Biol. 20, 262–271 (2018); Changede, R. et al. Nat. Mater. 18, 1366–1375 (2019); Oria, R. et al. Nature 552, 219–224 (2017).
Data exclusions	No data were excluded for data analysis.
Replication	All experiments were repeated independently at least two times with similar results.
Randomization	The experiments were not randomized.
Blinding	Blinding was not performed, as the protein sub-cellular distribution would clearly reveal the sample identity to investigators during most experiments. All data were collected automatically by instruments other than by human evaluation.

Reporting for specific materials, systems and methods

We require information from authors about some types of materials, experimental systems and methods used in many studies. Here, indicate whether each material, system or method listed is relevant to your study. If you are not sure if a list item applies to your research, read the appropriate section before selecting a response.

Materials & experimental systems

n/a	Involved in the study	
☐	☒	Antibodies
☐	☒	Eukaryotic cell lines
☒	☐	Palaeontology and archaeology
☒	☐	Animals and other organisms
☒	☐	Human research participants
☒	☐	Clinical data
☒	☐	Dual use research of concern

Methods

n/a	Involved in the study	
☒	☐	ChIP-seq
☒	☐	Flow cytometry
☒	☐	MRI-based neuroimaging

Antibodies

Antibodies used

Name Supplier Identifier Application Dilution
1.Rat anti-integrin β1 antibody (clone 9EG7) BD Biosciences Cat#550531 IF 1/100
2.Mouse anti-activated integrin β1 antibody (clone HUTS-4) Sigma-Aldrich Cat#MAB2079Z IF 1/100
3.Mouse anti-integrin αvβ5 antibody (clone P5H9) R&D Systems Cat#MAB2528 IF 1/100
4.Mouse anti-alpha adaptin antibody [AP6] Abcam Cat#ab2730 IF 1/250
5.Mouse anti-vinculin antibody (clone hVIN-1) Sigma-Aldrich Cat#V9131 IF 1/250
6.Mouse anti-paxillin antibody (clone 349/Paxillin) BD Biosciences Cat#610051 IF 1/250
7.Mouse anti-talin1 antibody (clone 8D4) Abcam Cat#ab157808 IF 1/100
8.Mouse Anti-Vitronectin/S-Protein antibody (clone VN58-1) Abcam Cat#ab13413 IF 1/250
9.Rabbit anti-FAK (phospho Y397) antibody (clone EP2160Y) Abcam Cat#ab81298 IF 1/250
10.Rabbit anti-Clathrin heavy chain antibody (Polyclonal) Abcam Cat#ab21679 IF 1/250
11.Rabbit anti-integrin β5 antibody (clone D24A5) Cell signaling technology Cat#3629S IF 1/250 and WB 1/1000
12.Rabbit anti-FCHo2 antibody (Polyclonal) Novus Biologicals Cat#NBP2-32694 WB 1/1000
13.Rabbit anti-GAPDH (clone 14C10) Cell signaling technology Cat#2118 WB 1/5000
14.Rabbit anti-GFP antibody (Polyclonal) Invitrogen Cat#A-11122 WB 1/1000
15.Mouse anti-mCherry antibody (clone GT857) Sigma-Aldrich Cat#SAB2702291 WB 1/1000
16.Alexa Fluor 488-conjugated goat anti-Mouse IgG (H+L) antibody Thermo Fisher Scientific Cat#A-11001 IF 1/500
17.Alexa Fluor 568-conjugated goat anti-Mouse IgG (H+L) antibody Thermo Fisher Scientific Cat#A-11004 IF 1/500
18.Alexa Fluor 488-conjugated goat anti-Rabbit IgG (H+L) antibody Thermo Fisher Scientific Cat#A-11034 IF 1/500
19.Texas Red-conjugated goat anti-Rabbit IgG (H+L) antibody Thermo Fisher Scientific Cat#T2767 IF 1/500
20.Alexa Fluor 647-conjugated goat anti-Rat IgG (H+L) antibody Thermo Fisher Scientific Cat#A-21247 IF 1/500

21.HRP-linked goat anti-Rabbit IgG (H+L) antibody Cell signaling technology Cat#7074 WB 1/1000
22.HRP-linked goat anti-Mouse IgG (H+L) antibody Cell signaling technology Cat#7076 WB 1/1000

Validation

The antibodies have been validated by the manufacturers and previous publications.
Details are available from the references below.
1.https://www.bdbiosciences.com/en-us/products/reagents/flow-cytometry-reagents/research-reagents/single-color-antibodies-ruo/purified-rat-anti-mouse-cd29.550531
2.https://www.sigmaaldrich.com/US/en/product/mm/mab2079z
3.https://www.rndsystems.com/products/human-integrin-alphavbeta5-antibody-p5h9_mab2528
4.https://www.abcam.com/products/primary-antibodies/alpha-adaptin-antibody-ap6-ab2730.html
5.https://www.sigmaaldrich.com/US/en/product/sigma/v9131
6.https://www.bdbiosciences.com/en-us/products/reagents/microscopy-imaging-reagents/immunofluorescence-reagents/purified-mouse-anti-paxillin.610051
7.https://www.abcam.com/products/primary-antibodies/talin-1-antibody-8d4-ab157808.html
8.https://www.citeab.com/antibodies/759488-ab13413-anti-vitronectin-s-protein-antibody-vn58-1
9.https://www.abcam.com/products/primary-antibodies/fak-phospho-y397-antibody-ep2160y-ab81298.html
10.https://www.abcam.com/products/primary-antibodies/clathrin-heavy-chain-antibody-ab21679.html
11.https://www.cellsignal.com/products/primary-antibodies/integrin-b5-d24a5-rabbit-mab/3629
12.https://www.novusbio.com/products/fcho2-antibody_nbp2-32694
13.https://www.cellsignal.com/products/primary-antibodies/gapdh-14c10-rabbit-mab/2118
14.https://www.thermofisher.com/antibody/product/GFP-Antibody-Polyclonal/A-11122
15.https://www.sigmaaldrich.com/US/en/product/sigma/sab2702291
16.https://www.thermofisher.com/antibody/product/Goat-anti-Mouse-IgG-H-L-Cross-Adsorbed-Secondary-Antibody-Polyclonal/A-11001
17.https://www.thermofisher.com/antibody/product/Goat-anti-Mouse-IgG-H-L-Cross-Adsorbed-Secondary-Antibody-Polyclonal/A-11004
18.https://www.thermofisher.com/antibody/product/Goat-anti-Rabbit-IgG-H-L-Highly-Cross-Adsorbed-Secondary-Antibody-Polyclonal/A-11034
19.https://www.thermofisher.com/antibody/product/Goat-anti-Rabbit-IgG-H-L-Cross-Adsorbed-Secondary-Antibody-Polyclonal/T-2767
20.https://www.thermofisher.com/antibody/product/Goat-anti-Rat-IgG-H-L-Cross-Adsorbed-Secondary-Antibody-Polyclonal/A-21247
21.https://www.cellsignal.com/products/secondary-antibodies/anti-rabbit-igg-hrp-linked-antibody/7074
22.https://www.cellsignal.com/products/secondary-antibodies/anti-mouse-igg-hrp-linked-antibody/7076

Eukaryotic cell lines

Policy information about cell lines

Cell line source(s)

U2OS (ATCC HTB-96™), A549 (ATCC CCL-185™), HeLa (ATCC CCL-2™), Mouse Embryonic Fibroblast (MEF) (ATCC CRL-2991™), IMR-90 lung fibroblast cells (ATCC® CCL-186™, a gift from Scott Dixon), HEK293T cells (ATCC® CRL-3216™), HT-1080 (ATCC CCL-121™), U-251MG (Sigma-Aldrich, 09063001), human mesenchymal stem cells (PCS-500-011™)， and MCF7 (ATCC HTB-22™)

Authentication

Cell line authentication was not performed. All cell lines were expanded from the original vials vendors provided and used for experiments within ten passages.

Mycoplasma contamination

All cell lines were tested negative for mycoplasma contamination by RT-PCR.

Commonly misidentified lines
(See ICLAC register)

We did not use commonly misidentified lines listed by ICLAC.
