# Supplementary figures and images for: Curved adhesions mediate cell attachment to soft matrix fibres in three dimensions
Source: Nat Cell Biol. 2023 Sep 28;25(10):1453–64. doi: 10.1038/s41556-023-01238-1 (PMC10567576; doi:10.1038/s41556-023-01238-1)

**Fig. 4n**

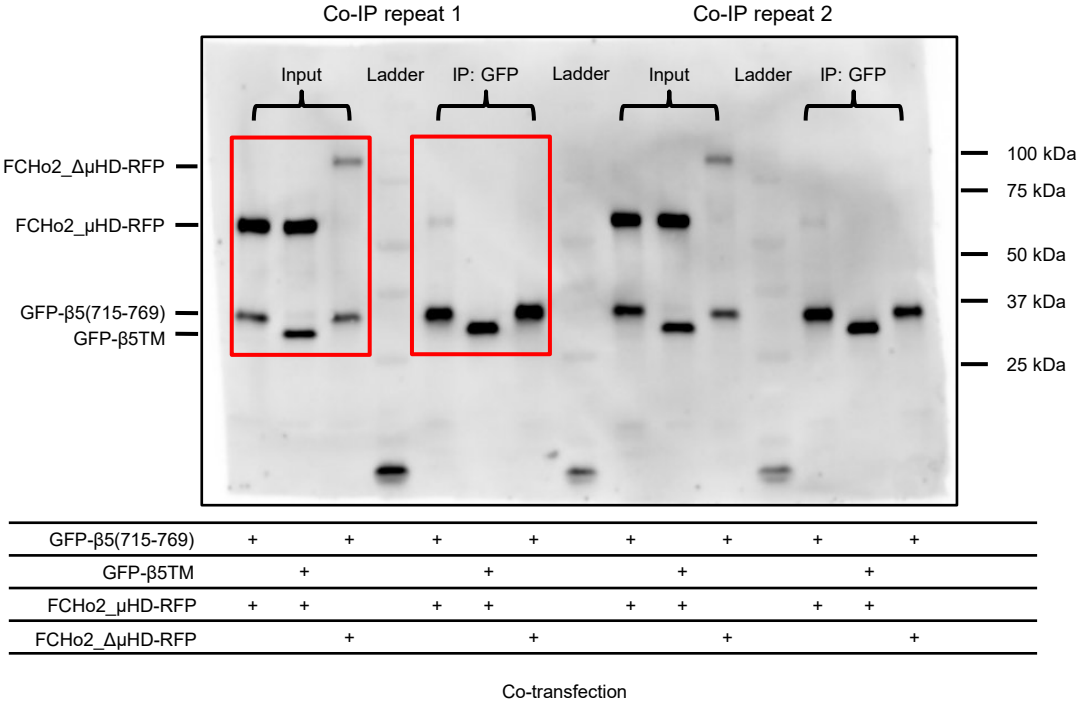

Supplement: Supplementary file 14 — Unprocessed western blots. [file 41556_2023_1238_MOESM14_ESM.pdf]
