# Supplementary material for: Curved adhesions mediate cell attachment to soft matrix fibres in three dimensions
Source: Nat Cell Biol. 2023 Sep 28;25(10):1453–64. doi: 10.1038/s41556-023-01238-1 (PMC10567576; doi:10.1038/s41556-023-01238-1)
Supplement: Supplementary file 17 — Unprocessed western blots. [file 41556_2023_1238_MOESM17_ESM.pdf]

Fig. 6b

FCHo2 knockdown

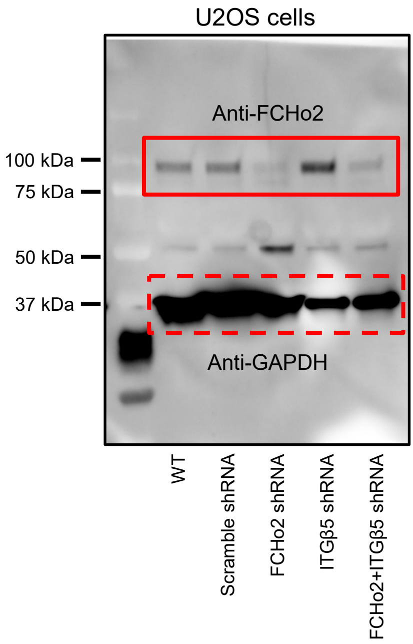

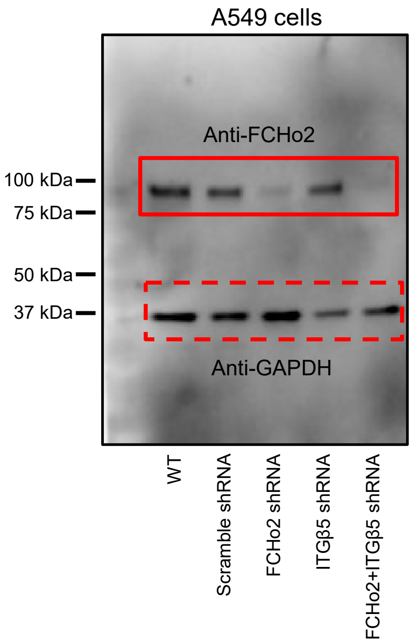

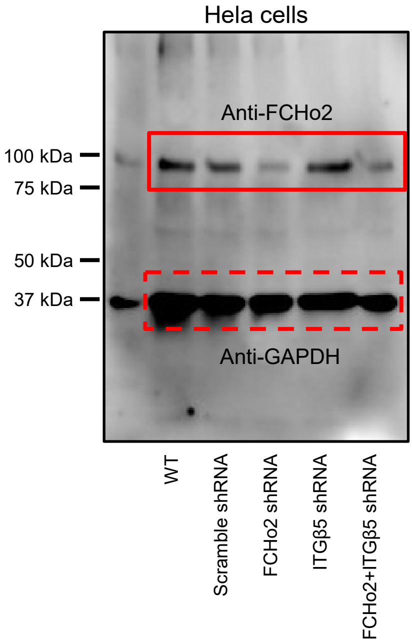

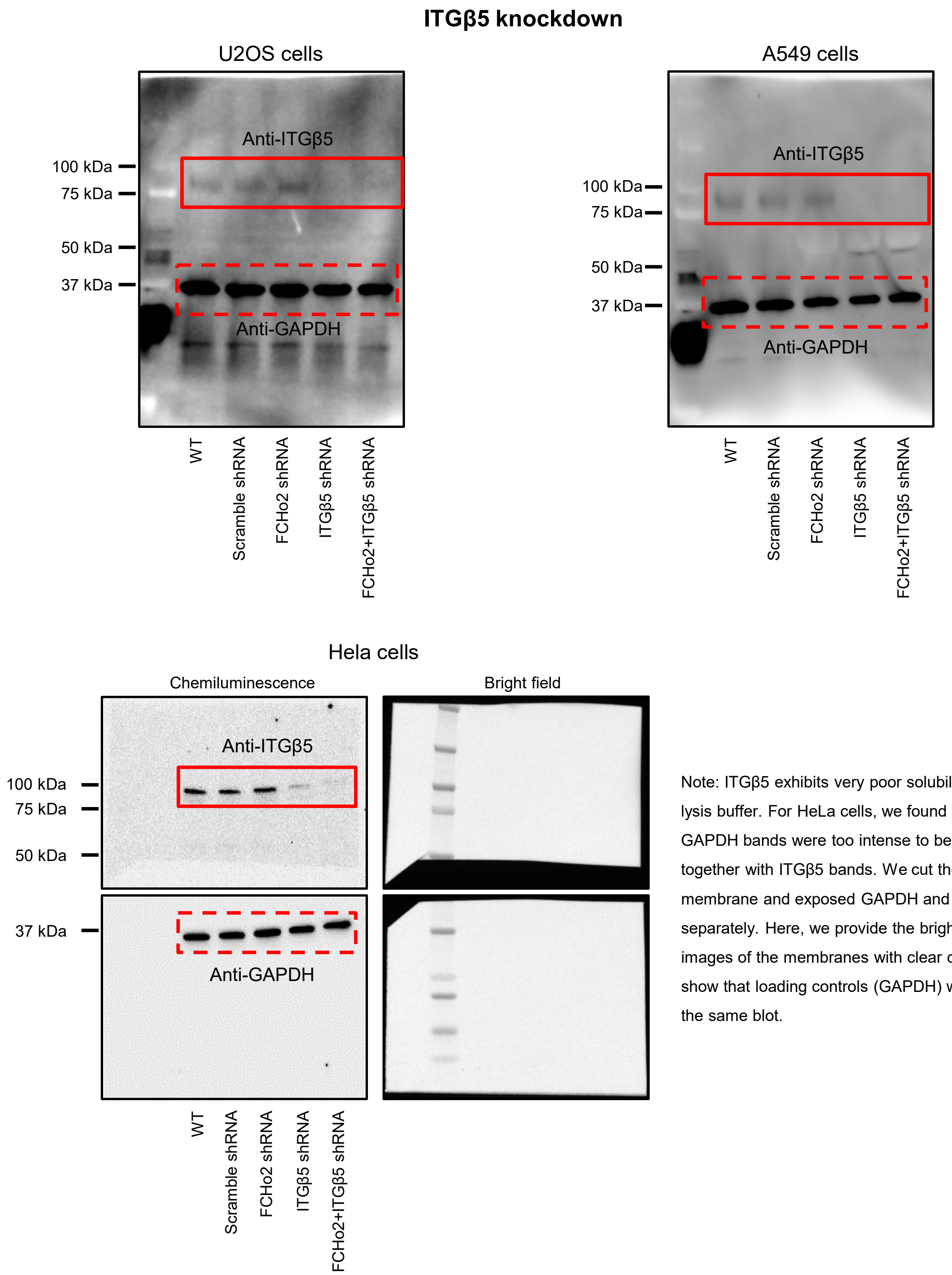

Note: ITGβ5 exhibits very poor solubility in RIPA lysis buffer. For HeLa cells, we found that GAPDH bands were too intense to be exposed together with ITGβ5 bands. We cut the membrane and exposed GAPDH and ITGβ5 separately. Here, we provide the bright field images of the membranes with clear cut marks to show that loading controls (GAPDH) were run on the same blot.
